# Confidence regions for repeated measures ANOVA power curves based on estimated covariance

**DOI:** 10.1186/1471-2288-13-57

**Published:** 2013-04-15

**Authors:** Matthew J Gribbin, Yueh-Yun Chi, Paul W Stewart, Keith E Muller

**Affiliations:** 1 Department of Biostatistics, MedImmune, Gaithersburg, MD, USA; 2 Department of Biostatistics, University of Florida, Gainesville; 3 Department of Biostatistics, University of North Carolina, Chapel Hill, NC, USA; 4 Department of Health Outcomes and Policy, University of Florida, Gainesville, FL, USA

**Keywords:** Sample size, Replication study, Study planning, Univariate approach, UNIREP

## Abstract

**Background:**

Using covariance or mean estimates from previous data introduces randomness into each power value in a power curve. Creating confidence intervals about the power estimates improves study planning by allowing scientists to account for the uncertainty in the power estimates. Driving examples arise in many imaging applications.

**Methods:**

We use both analytical and Monte Carlo simulation methods. Our analytical derivations apply to power for tests with the univariate approach to repeated measures (UNIREP). Approximate confidence intervals and regions for power based on an estimated covariance matrix and fixed means are described. Extensive simulations are used to examine the properties of the approximations.

**Results:**

Closed-form expressions are given for approximate power and confidence intervals and regions. Monte Carlo simulations support the accuracy of the approximations for practical ranges of sample size, rank of the design matrix, error degrees of freedom, and the amount of deviation from sphericity. The new methods provide accurate coverage probabilities for all four UNIREP tests, even for small sample sizes. Accuracy is higher for higher power values than for lower power values, making the methods especially useful in practical research conditions. The new techniques allow the plotting of power confidence regions around an estimated power curve, an approach that has been well received by researchers. Free software makes the new methods readily available.

**Conclusions:**

The new techniques allow a convenient way to account for the uncertainty of using an estimated covariance matrix in choosing a sample size for a repeated measures ANOVA design. Medical imaging and many other types of healthcare research often use repeated measures ANOVA.

## Background

### Motivation

Computing power for a linear model involving repeated measures requires specifying a set of means and a covariance matrix. Scientists usually feel comfortable specifying a pattern of means that corresponds to a difference of clinical or scientific importance. However, specifying plausible variance and covariance values usually requires estimates from a previous study.

Using data from a previous study to estimate the covariance matrix makes the power value a random variable. Kraemer, et al. [1] noted that if the estimated variance is too small, i.e., when the pilot study is overly favorable, power will be over-estimated. Conversely, if the estimated variance is too large (pilot study overly conservative), power will be under-estimated. Maxwell [2] conducted simulations to illustrate the amount of bias that can occur. Taylor and Muller [3] and Muller and Pasour [4] derived exact distributions of noncentrality and power in univariate linear models based on all combinations of estimated variance and means. The results account for power computed conditional on a previous result being significant, or conditional on a previous result being non-significant. The former creates optimistic bias, while the latter creates pessimistic bias.

Providing confidence intervals to account for the uncertainty inherent in the random power values would be useful for study planning. For example, a lower bound for power would allow stating that a test has power of at least “*P*” to detect an effect, with a specified confidence. A confidence region for a power curve would be even more informative.

Medical imaging research motivated the work here because it often generates the type of complete data that can be handled with the univariate approach to repeated measures (UNIREP). Muller, et al. [5] reviewed the advantages gained by being able to use the UNIREP model, a special case of the general linear mixed model. The same authors described accurate and convenient power approximations for UNIREP analysis. The four UNIREP tests, Box conservative, Geisser-Greenhouse, Huynh-Feldt, and the uncorrected, all use the same test statistic. For data analysis, UNIREP tests differ only by their respective degrees of freedom due to different degrees of freedom multipliers, which measure sphericity in the error covariance for the hypothesis variables. Muller and Stewart [6] provided detailed discussion of the basic theory for both the null and non-null cases. Earlier work detailed basic UNIREP theory. Box [7,8], Geisser and Greenhouse [9,10] and Huynh and Feldt [11] gave null results. Davies [12] and Muller and Barton [13,14] treated the non-null case.

Browne [15] evaluated the impact of using a pilot study to estimate the variance for a *t*-test. More generally, Taylor and Muller [16] demonstrated how to construct exact power confidence intervals for the general linear univariate model for a data-estimated variance and fixed means. The same authors also generalized the result to provide an exact confidence region around a power curve. Parallel results for the UNIREP setting would be equally useful. We generalize the methods in Taylor and Muller [16] to UNIREP tests for repeated measures. We use analytic and simulation results to demonstrate that the techniques allow computing approximate confidence intervals and regions for power with good accuracy for the UNIREP tests, based on an estimated covariance matrix and fixed means.

### Existing results

A vector ***z***, (*n*×1), is lower case bold. A matrix, ***z***, is upper case bold with transpose ***Z***^′^, inverse ***Z***^−1^ and generalized inverse ***Z***^−^. Also, ***1***_*n*_ is an (*n*×1) vector of 1’s and ***I***_*n*_ is an (*n*×*n*) identity matrix. A diagonal matrix with (*i*,*i*) element *z*_*i*_ is written Dg (***z***). The expected value, variance, and trace are E(***Z***), $V\left(Z\right)$, and tr(***z***), respectively. Throughout, *Z*∼*χ*^2^(*ν*,*ω*) indicates that *Z* has a noncentral chi-square distribution with *ν* degrees of freedom and noncentrality *ω*, while *Z*∼*χ*^2^(*ν*) indicates a central distribution. Similarly, *Z*∼*F*(*ν*_1_,*ν*_2_,*ω*) indicates *X* has a noncentral *F* distribution with *ν*_1_ numerator and *ν*_2_ denominator degrees of freedom, and noncentrality *ω* with cumulative distribution function *F*_*F*_(*ν*_1_,*ν*_2_,*ω*). A central *F* is written *Z*∼*F*(*ν*_1_,*ν*_2_) with quantile *q* indicated $F_{F}^{-1}\left(q;\nu_{1},\nu_{2}\right)$. Writing $z∼N_{p}\left(\mu ,\Sigma \right)$ indicates ***z*** (*p*×1) is Gaussian with mean ***μ*** and covariance **Σ** (*p*×*p*). If ***z*** (*N*×*p*) has independent rows and ${\left[{\text{row}}_{i}\left(Z\right)\right]}^{\prime }∼N_{p}\left(\mu_{i},\Sigma \right)$, then $S=Z^{\prime }Z∼W_{p}\left(N,\Sigma ,\Omega \right)$ indicates ***S*** follows a Wishart distribution with *N* degrees of freedom, covariance **Σ**, and noncentrality ***Ω***=E(***Z***^′^)E(***Z***)**Σ**^−1^.

The general linear multivariate model,

(1) $$\begin{array}{c} \;Y\;=\;\mathrm{XB}\;+\;E\;\text{,}\\ \left(N\times p\right)\;\left(N\times q\times p\right)\;\left(N\times p\right) \end{array}$$

assumes *N* independent rows and ${\left[{\text{row}}_{i}\left(Y\right)\right]}^{\prime }∼N_{p}\left({\left[{\text{row}}_{i}\left(X\right)B\right]}^{\prime },\Sigma \right)$. In the model, ***X*** is the fixed, known design matrix with 1≤rank(*X*)≤*q*, and ***B*** contains fixed, unknown regression coefficients. For repeated measures ANOVA, one-group designs have rank (***X***)=1, and two-group comparisons have rank (***X***)=2. The associated general linear hypothesis is

(2) $$\begin{array}{l} H_{0}:\;\Theta \;=\;\mathrm{CBU}\;=\;\Theta_{0}\;\text{,}\\ \;\left(a\times b\right)\;\left(a\times q\right)\left(q\times p\right)\left(p\times b\right)\;\left(a\times b\right) \end{array}$$

suchthat ***C***definesthebetween-subject effects(rank *a*) while ***U*** defines the within-subject effects (rank *b*). Requiring estimable **Θ** and full rank {***C***,***U***} ensure a testable hypothesis. Appropriate selections of the contrast matrices (***C*** and ***U***) and null matrix (**Θ**_0_) allows testing important one-degree-of-freedom parameters, such as the difference between two means, or a comparison of two trends.

For ***M*** = ***C***(***X***^′^***X***)^−^***C***^′^, unscaled noncentrality is ***Δ*** = (**Θ**−**Θ**_0_)^′^***M***^−1^(**Θ**−**Θ**_0_), scaled noncentrality is $\Omega ={\Delta \Sigma }_{*}^{-1}$. Here **Σ**_∗_=***U***^′^***ΣU***=***Υ***Dg (***λ***)***Υ***^′^ is the covariance matrix among the hypothesis variables, with ***ΥΥ***^′^=***Υ***^′^***Υ***=***I***_*b*_, and ***λ***={*λ*_*k*_} the eigenvalues. Estimates are $\tilde{B}={\left(X^{\prime }X\right)}^{-}X^{\prime }Y$ and $\hat{\Sigma }=Y^{\prime }\left[I-{\left(X^{\prime }X\right)}^{-}X^{\prime }\right]Y/\nu_{e}$, with *ν*_*e*_=*N*−rank(***X***), the error degrees of freedom. Furthermore, $\hat{\Theta }=C\tilde{B}U$, $\hat{\Delta }={\left(\hat{\Theta }-\Theta_{0}\right)}^{\prime }M^{-1}(\hat{\Theta }-\Theta_{0})∼W_{b}\left(a,\Sigma_{*},\Omega \right)$ and ${\hat{\Sigma }}_{*}=U^{\prime }\hat{\Sigma }U$, with $\nu_{e}{\hat{\Sigma }}_{*}∼W_{b}\left(\nu_{e},\Sigma_{*}\right)$. The sum of squares hypothesis matrix is $S_{H}=\hat{\Delta }$ and the sum of squares error matrix is $S_{E}=\nu_{e}{\hat{\Sigma }}_{*}$, which are independent of one another. The notation follows that in Muller and Stewart [6]. Additional notation is in Appendix A: Additional notation.

The univariate approach to repeated measures can be expressed in terms of the general linear multivariate model. The Box conservative (Box), the Geisser-Greenhouse (GG), the Huynh-Feldt (HF), and the uncorrected (Un) UNIREP tests use the same test statistic,

(3) $$T_{u}=\frac{/)\text{tr}(\hat{\Delta })a}{\text{tr}({\hat{\Sigma }}_{*})}\text{,}$$

and a central *F* distribution to approximate the null distribution of *T*_*u*_,

(4) $$\mathrm{Pr}\left\{T_{u}\leq t\right\}\approx F_{F}\left(t;\mathrm{ab∊},\nu_{e}\mathrm{b∊},0\right).$$

The sphericity parameter, *∊*=tr^2^(**Σ**_∗_)/[*b*tr(**Σ**∗2)], quantifies the spread of population eigenvalues and is used to discount the degrees of freedom. The term *sphericity* reflects the fact that uncorrelated Gaussian variables with equal variances in three dimensions have a spherical scattergram. The eigenvalues of **Σ**_∗_ are the variances of the (uncorrelated) principal components of the hypothesis response variables. Perfect sphericity requires *∊* = 1, which occurs with all eigenvalues equal. Minimal sphericity has *∊*=1/*b*, which occurs with one nonzero eigenvalue. Other patterns of **Σ**_∗_ have 1/*b*<*∊*<1.

The Box conservative test uses the fixed, lower bound of *∊*, while the uncorrected test uses the fixed, upper bound of *∊*. With sphericity (*∊*=1), the uncorrected test is exact and uniformly most powerful (among similarly invariant tests). The Geisser-Greenhouse and Huynh-Feldt tests use the observed data to estimate *∊*. The Geisser-Greenhouse estimator, $\hat{∊}={\text{tr}}^{2}({\hat{\Sigma }}_{*})/b\text{tr}({\hat{\Sigma }}_{*}^{2})$, is the maximum likelihood (ML) estimator. The Huynh-Feldt estimator, $\tilde{∊}=\left(\text{Nb}\hat{∊}-2\right)/\left[b\left(\nu_{e}-b\hat{∊}\right)\right]$ was proposed as the ratio of two unbiased estimators. Their claim holds only for the special case of rank (***X***)=1. Lecoutre [17] provided a more general form. In turn, Gribbin [18] and Chi et al. [19] described a rank-adjusted approximately unbiased estimator, ${\tilde{∊}}_{r}=\left[\left(\nu_{e}+1\right)b\hat{∊}-2\right]/\left[b\left(\nu_{e}-b\hat{∊}\right)\right]$, which applies to any general linear multivariate model. The rank-adjusted power approximation was shown through simulations to approximate observed mean power values as well as, or better than, the Huynh-Feldt power approximation (Chi et al. [19]). Only the rank-adjusted Huynh-Feldt estimator will be considered in the remainder of the paper.

Although the four UNIREP tests all use the same test statistic, they each use a different measure of sphericity, here indicated *e*. For data analysis, all four tests use a critical value $q(e)=F_{F}^{-1}\left(1-\alpha ,\nu_{1}e,\nu_{2}e\right)$. Here *ν*_1_=*ab* and *ν*_2_=*bν*_*e*_. The Box test uses *e*=1/*b*, the GG test uses $e=\hat{∊}$, HF uses $e=\tilde{∊}$, and the uncorrected test uses *e*=1. The p-value is then computed, for observed test statistic *t*, as *p*=1−*F*(*t*,*ν*_1_*e*,*ν*_2_*e*). In all cases $1/b\leq \hat{∊}\leq \tilde{∊}\leq 1$. In turn, the *p*-values always have the reverse order, with the Box *p*-value being largest, and the uncorrected being smallest.

Muller et al. [5] showed that the distribution function of the UNIREP test statistic can be expressed exactly in terms of the distribution function of the sum of *b* positively and *b* negatively weighted independent chi-squares, namely *y*_*kh*_∼*χ*^2^(*a*,*ω*_*k*_) and *y*_*ke*_∼*χ*^2^(*ν*_*e*_),

(5) $$\begin{array}{lll} \mathrm{Pr}\left\{T_{u}\leq t\right\} & =\mathrm{Pr}\left\{\frac{\text{tr}(\hat{\Delta })a\left(\right)}{\text{tr}({\hat{\Sigma }}_{*})}\leq t\right\}\; & \;\\ & =\mathrm{Pr}\left\{\overset{b}{\underset{k=1}{\sum }}\lambda_{k}y_{\text{kh}}-\left(\text{ta}/\nu_{e}\right)\overset{b}{\underset{k=1}{\sum }}\lambda_{k}y_{\text{ke}}\leq 0\right\}\; \end{array}$$

Muller et al. [5] also reported accurate *F* approximations of the form

(6) $$\begin{array}{lll} \mathrm{Pr}\left\{T_{u}\leq t\right\} & \approx \mathrm{Pr}\left\{\frac{\lambda_{*1}y_{*1}/(\text{ab})}{\lambda_{*2}y_{*2}/\left(b\nu_{e}\right)}\leq t\right\}\; & \;\\ & =F_{F}\left(t\frac{\lambda_{*2}}{\lambda_{*1}}\frac{\text{ab}}{\nu_{*1}}\frac{\nu_{*2}}{b\nu_{e}};\nu_{*1},\nu_{*2},\omega_{*}\right)\text{.}\; \end{array}$$

Here, *y*_∗1_∼*χ*^2^(*ν*_∗1_,*ω*_∗_), *y*_∗2_∼*χ*^2^(*ν*_∗2_), $\text{tr}(\hat{\Delta })\approx \lambda_{*1}y_{*1}$ and $\nu_{e}\text{tr}({\hat{\Sigma }}_{*})\approx \lambda_{*2}y_{*2}$. Parameters *λ*_∗1_, *ν*_∗1_, *ω*_∗_, *λ*_∗2_, and *ν*_∗2_ are defined in Appendix A: Additional notation. Power analysis involves {*λ*_*k*_} and {*ω*_*k*_}, with

(7) $$\omega_{k}=\upsilon_{k}^{\prime }{\Delta \upsilon }_{k}/\lambda_{k}\text{,}$$

the diagonal elements of the scaled noncentrality, ***Ω***_∗_=***Υ***^′^***ΔΥ***Dg (***λ***)^−1^=***Δ***_∗_Dg (***λ***)^−1^.

## Methods

### Estimating approximate UNIREP power with estimated covariance and fixed means

By extending results in Muller et al. [5], the following lemma helps simplify the *F* approximations. Appendix B: Supporting lemmas and proofs contains all proofs.

#### Lemma 1

The constant in the critical value of the UNIREP test statistic approximation introduced by Muller et al. [5] is equal to 1,

(8) $$\frac{\lambda_{*2}}{\lambda_{*1}}\frac{\text{ab}}{\nu_{*1}}\frac{\nu_{*2}}{b\nu_{e}}=1\text{.}$$

Thus,

(9) $$\begin{array}{lll} \mathrm{Pr}\left\{T_{u}\leq t\right\} & \approx \mathrm{Pr}\left\{\frac{\lambda_{*1}y_{*1}/(\text{ab})}{\lambda_{*2}y_{*2}/\left(b\nu_{e}\right)}\leq t\right\}\; & \;\\ & =F_{F}\left(t;\nu_{*1},\nu_{*2},\omega_{*}\right)\text{.}\; \end{array}$$

For known covariance and means, the power approximations for the Box, Geisser-Greenhouse, rank-adjusted Huynh-Feldt, and uncorrected tests are all of the form

(10) $$\;P=1-F_{F}\;\left[\;F_{F}^{-1}\;\left(1\;-\alpha ;e_{1}\;\cdot \text{ab},e_{2}\;\cdot b\nu_{e}\right);e_{3}\cdot \text{ab},e_{4}\cdot \nu_{e}b,\frac{\text{tr}\left(\Delta \right)}{\bar{\lambda }/e_{5}}\right]\text{.}$$

Here, $\bar{\lambda }$ is equal to tr(**Σ**_∗_)/*b* with *b* equal to the rank of **Σ**_∗_, and $\omega_{*}=\text{tr}\left(\Delta \right)/\left(\bar{\lambda }/e_{5}\right)$. Table 1 contains values for *e*_1_ through *e*_5_ for the four UNIREP tests when ${∊}_{d}=∊={\text{tr}}^{2}(\Sigma_{*})/b\text{tr}(\Sigma_{*}^{2})$, and ${∊}_{n}=\left[{\text{tr}}^{2}(\Sigma_{*})+2\text{tr}(\Sigma_{*})\text{tr}\left(\Delta /a\right)\right]\left\{b\left[\text{tr}(\Sigma_{*}^{2})+2\text{tr}(\Sigma_{*}\Delta /a)\right]\right\}$. The expressions for *∊*_*d*_ and *∊*_*n*_ were derived using the properties described in Lemma B.1.

**Table 1:** Sphericity multipliers for UNIREP power approximations for fixed means

		**Multipliers**				
**Covariance**	**Test**	***e***_**1**_	***e***_**2**_	***e***_**3**_	***e***_**4**_	***e***_**5**_
**Σ**_∗_(known)	Un	1	1	*∊*_*n*_	*∊*_*d*_	*∊*_*n*_
	HF	$\text{E}\left(\tilde{∊}\right)$	$\text{E}\left(\tilde{∊}\right)$	*∊*_*n*_	*∊*_*d*_	*∊*_*n*_
	GG	$\text{E}\left(\hat{∊}\right)$	$\text{E}\left(\hat{∊}\right)$	*∊*_*n*_	*∊*_*d*_	*∊*_*n*_
	Box	1/*b*	1/*b*	*∊*_*n*_	*∊*_*d*_	*∊*_*n*_
${\hat{\Sigma }}_{*}$ (estimated)	Un	1	1	${\tilde{∊}}_{n}$	${\hat{∊}}_{d}$	${\tilde{∊}}_{n}$
	HF	${\tilde{∊}}_{r}$	${\tilde{∊}}_{r}$	${\tilde{∊}}_{n}$	${\hat{∊}}_{d}$	${\tilde{∊}}_{n}$
	GG	${\hat{∊}}_{d}$	${\hat{∊}}_{d}$	${\tilde{∊}}_{n}$	${\hat{∊}}_{d}$	${\tilde{∊}}_{n}$
	Box	1/*b*	1/*b*	${\tilde{∊}}_{n}$	${\hat{∊}}_{d}$	${\tilde{∊}}_{n}$

In practice, some elements of $\left\{e_{1},e_{2},e_{3},e_{4},e_{5},\text{tr}(\Delta ),\bar{\lambda }\right\}$ may be estimated and hence random. The random elements imply random power values, as with estimated covariance and fixed means, $\left\{{\hat{\Sigma }}_{*},\Delta \right\}$, for ${\hat{\Sigma }}_{*}\;=\;{\hat{E}}^{\prime }\hat{E}/\nu_{\text{est}}$, the unbiased restricted maximum likelihood (REML) estimator. A distinction must be carefully maintained between the estimation study and target study. The estimation study provides the covariance estimate and has sample size *N*_est_, design matrix rank of rank (***X***_est_), and *ν*_est_=*N*_est_−rank(***X***_est_) degrees of freedom. The target study for which power is desired has sample size *N*, rank (***X***) and *ν*_*e*_=*N*−rank(***X***) degrees of freedom.

The ML estimator from the Geisser-Greenhouse test, $\hat{∊}={\text{tr}}^{2}({\hat{\Sigma }}_{*})/[b\text{tr}({\hat{\Sigma }}_{*}^{2})]$, is an obvious estimator for the target study’s *∊*. For power analysis, a parallel estimator is available for *∊*_*n*_:

(11) $${\hat{∊}}_{n}=\frac{{\text{tr}}^{2}({\hat{\Sigma }}_{*})+2\text{tr}({\hat{\Sigma }}_{*})\text{tr}\left(\Delta /a\right)}{b\left[\text{tr}({\hat{\Sigma }}_{*}^{2})+2\text{tr}({\hat{\Sigma }}_{*}\Delta /a)\right]}.$$

A better choice, given in the following lemma, uses a ratio of unbiased estimators. The result generalizes the rank-adjusted Huynh-Feldt estimator for data analysis. Appendix B: Supporting lemmas and proofs has derivations of moments as well as all proofs.

#### Lemma 2

For the non-null case, a ratio estimating *∊*_*n*_ in terms of correlated, but unbiased, estimators is [b]

(12) $$\;\;\;{\tilde{∊}}_{n}=\frac{\nu_{\text{est}}\left(\nu_{\text{est}}+1\right){\text{tr}}^{2}({\hat{\Sigma }}_{*})-2\nu_{\text{est}}\text{tr}({\hat{\Sigma }}_{*}^{2})+2\left[\nu_{\text{est}}\left(\nu_{\text{est}}+1\right)-2\right]\text{tr}({\hat{\Sigma }}_{*})\text{tr}\left(\Delta /a\right)}{b\left\{\nu_{\text{est}}^{2}\text{tr}({\hat{\Sigma }}_{*}^{2})-\nu_{\text{est}}{\text{tr}}^{2}({\hat{\Sigma }}_{*})+2\left[\nu_{\text{est}}\left(\nu_{\text{est}}+1\right)-2\right]\text{tr}({\hat{\Sigma }}_{*}\Delta /a)\right\}}\text{.}$$

The corresponding estimator for the null case is ${\tilde{∊}}_{r}=\left[\left(\nu_{\text{est}}+1\right)b\hat{∊}-2\right]/\left[b\left(\nu_{\text{est}}-b\hat{∊}\right)\right]$, the rank-adjusted Huynh-Feldt sphericity estimator.

For estimated covariance and fixed means, approximate estimated UNIREP power is

(13) $$\;P=1-F_{F}\left[\;F_{F}^{-1}\left(1\;-\alpha ;e_{1}\;\cdot \text{ab},e_{2}\;\cdot b\nu_{e}\right);e_{3}\;\cdot \text{ab},e_{4}\;\cdot \nu_{e}b,\frac{\text{tr}\left(\Delta \right)}{\bar{\hat{\lambda }}/e_{5}}\right]\text{,}$$

with $\bar{\hat{\lambda }}=\text{tr}({\hat{\Sigma }}_{*})/b$, and *e*_1_ through *e*_5_ estimated if unknown (Table 1). Nearly every combination of ${\hat{∊}}_{n}$, ${\tilde{∊}}_{n}$, ${\hat{∊}}_{d}$, ${\tilde{∊}}_{r}$, 1 and 1/*b* was examined for each UNIREP test for the wide range of simulations discussed in Muller et al. [5]. The values chosen provided the most accurate results. In retrospect, they are natural choices as well.

### Approximate UNIREP power confidence intervals

The solution to the UNIREP problem parallels the solution to the univariate problem in Taylor and Muller [16]. The methods apply to any general linear hypothesis, including one degree-of-freedom contrasts, such as pair-wise group comparisons and differences in linear trend between two groups. Tests giving scalar secondary parameters are also common for one-group designs and two-group comparisons. For known covariance and means, *e*_5_ is defined to be *∊*_*n*_ (Table 1), and the noncentrality in equation 9 is $\omega_{*}=\left[\text{tr}\left(\Delta \right)\right]/(\bar{\lambda }/{∊}_{n})=\left[\text{tr}\left(\Delta \right)\right]/\lambda_{*1}$ with $\bar{\lambda }=\text{tr}(\Sigma_{*})/b$ and $\lambda_{*1}=\bar{\lambda }/{∊}_{n}$. For ${∊}_{n}=\left[{\text{tr}}^{2}(\Sigma_{*})+2\text{tr}(\Sigma_{*})\text{tr}\left(\Delta /a\right)\right]\left\{b\left[\text{tr}(\Sigma_{*}^{2})+2\text{tr}(\Sigma_{*}\Delta /a)\right]\right\}$. Therefore, it follows that

(14) $$\omega_{*}=\text{tr}\left(\Delta \right)\cdot \frac{\text{tr}\left(\Sigma_{*}\right)+2\text{tr}\left(\Delta /a\right)}{\text{tr}(\Sigma_{*}^{2})+2\text{tr}\left({\Delta \Sigma }_{*}/a\right)}\text{.}$$

For estimated covariance and fixed means, a ratio involving one biased and two unbiased estimators (Lemma B.2) for estimating *λ*_∗1_ may be written as

(15) $${\tilde{\lambda }}_{*1}=\frac{\text{tr}({\hat{\Sigma }}_{*}^{2})+2\text{tr}(\Delta {\hat{\Sigma }}_{*}/a)}{\text{tr}({\hat{\Sigma }}_{*})+2\text{tr}\left(\Delta /a\right)}\text{.}$$

In parallel to the univariate setting, the distribution of ${\tilde{\lambda }}_{*1}$ can be approximated with a Satterthwaite approximation: ${\tilde{\lambda }}_{*1}\nu_{*}/\lambda_{*1}∼\chi^{2}\left(\nu_{*}\right)$ with $\nu_{*}=\left(b\nu_{\text{est}}\right)\cdot {\hat{∊}}_{d}/{\tilde{∊}}_{n}$. Lower and upper tail probabilities, *α*_*L*_ and *α*_*U*_, respectively, define the confidence coefficient, *p*_*CL*_=1−*α*_*L*_−*α*_*U*_. Also, $c_{\mathrm{\alpha L}}=F_{\chi^{2}}^{-1}\left(\alpha_{L};\nu_{*}\right)$ and $c_{\mathrm{\alpha U}}=F_{\chi^{2}}^{-1}\left(1-\alpha_{U};\nu_{*}\right)$. Approximate confidence limits for the noncentrality may be calculated using the following:

(16) $$\begin{array}{l} \mathrm{Pr}\left\{c_{\mathrm{\alpha L}}<\frac{{\tilde{\lambda }}_{*1}\nu_{*}}{\lambda_{*1}}<c_{\mathrm{\alpha U}}\right\}\;\approx p_{\text{CL}}\; \end{array}$$

(17) $$\begin{array}{l} \mathrm{Pr}\left\{\frac{c_{\mathrm{\alpha L}}}{{\tilde{\lambda }}_{*1}\nu_{*}}<\frac{1}{\lambda_{*1}}<\frac{c_{\mathrm{\alpha U}}}{{\tilde{\lambda }}_{*1}\nu_{*}}\right\}\;\approx p_{\text{CL}}\; \end{array}$$

(18) $$\begin{array}{l} \mathrm{Pr}\left\{\frac{\text{tr}\left(\Delta \right)c_{\mathrm{\alpha L}}}{{\tilde{\lambda }}_{*1}\nu_{*}}<\frac{\text{tr}\left(\Delta \right)}{\lambda_{*1}}<\frac{\text{tr}\left(\Delta \right)c_{\mathrm{\alpha U}}}{{\tilde{\lambda }}_{*1}\nu_{*}}\right\}\;\approx p_{\text{CL}}\; \end{array}$$

(19) $$\begin{array}{l} \mathrm{Pr}\left\{\frac{\text{tr}\left(\Delta \right)c_{\mathrm{\alpha L}}}{{\tilde{\lambda }}_{*1}\nu_{*}}<\omega_{*}<\frac{\text{tr}\left(\Delta \right)c_{\mathrm{\alpha U}}}{{\tilde{\lambda }}_{*1}\nu_{*}}\right\}\;\approx p_{\text{CL}}\text{.}\; \end{array}$$

Approximate lower and upper bounds are therefore ${\tilde{\omega }}_{*L}=\text{tr}\left(\Delta \right)c_{\mathrm{\alpha L}}/{\tilde{\lambda }}_{*1}\nu_{*}$ and ${\tilde{\omega }}_{*U}\text{tr}\left(\Delta \right)c_{\mathrm{\alpha U}}/{\tilde{\lambda }}_{*1}\nu_{*}$. The strict monotone dependence of the noncentral *F* function on the noncentrality ensures an approximate confidence interval for power. Lower and upper bounds on power are, with *e*_1_ through *e*_4_ defined in Table 1 for ${\hat{\Sigma }}_{*}$,

(20) $$\;{\tilde{P}}_{L}=1-F_{F}\left[F_{F}^{-1}\left(1-\alpha ;e_{1}\cdot \text{ab},e_{2}\cdot b\nu_{e}\right);e_{3}\cdot \text{ab},e_{4}\cdot \nu_{e}b,{\tilde{\omega }}_{*L}\right]$$

and

(21) $$\;{\tilde{P}}_{U}=1-F_{F}\left[F_{F}^{-1}\left(1-\alpha ;e_{1}\;\cdot \text{ab},e_{2}\;\cdot b\nu_{e}\right);e_{3}\cdot \text{ab},e_{4}\cdot \nu_{e}b,{\tilde{\omega }}_{*U}\right]\text{.}$$

Taylor and Muller [16] recommended one-sided power confidence intervals by noting that “the change from a one-sided to a two-sided confidence interval has little effect on the upper bound, but a large effect on the lower bound”. Muller and Fetterman [20] provided examples of a one-sided power confidence interval in the univariate case.

### Approximate UNRIEP power confidence regions for power curves

The new methods allow calculating a confidence interval for a single power value. The logic of a proof in Taylor and Muller ([16] equations 2.1-2.13 and surrounding text) guarantees that accurate confidence regions are provided by the point-wise calculations. The proof may be sketched for the present setting as follows. Equations 14-21 establish the validity of the approximate confidence interval for a particular alternative hypothesis, as specified by the scalar constant tr(***Δ***). The randomness in the noncentrality arises from a scalar random variable, ${\tilde{\lambda }}_{*1}$, analogous to a variance. Equation 19 describes a single event with a specified probability. The inequality defining the event, and the associated probability, do not change for different values of the scalar constant tr(***Δ***). The smooth and strictly monotone dependence of power on the noncentrality ensures the validity of equations 20-21. The proof is completed by noting that the monotonicity extends the simultaneity property to the power confidence region.

**Figure 1:**
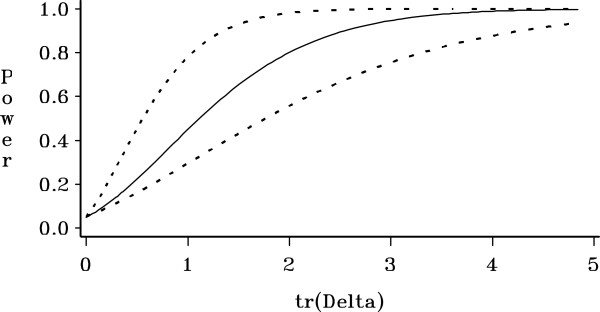
**Approximate 95% confidence region for predicted power of the rank-adjusted Huynh-Feldt test of interaction over tr(*Δ*) with *N = 10 *and population *∊=0.720 *for conditions described in Section ‘Simulation 1 with rank *(X) = 1 *(one-group repeated measures ANOVA)**’.

Figure 1 gives an example plot of approximate power confidence regions surrounding the predicted power curve for the rank-adjusted Huynh-Feldt test for *∊*=0.720. Graphical representations such as Figure 1 help researchers accurately recognize the amount of uncertainty in their power calculation, and lead to better decisions about design.

In some cases scientists prefer to consider sample size as a function of the pattern of mean differences. The theory already presented allows plotting sample size as a function of mean difference, albeit with a shift in algorithm. The power function must be numerically inverted to solve for the sample size desired. Taylor and Muller [16] outlined the steps of algorithm needed for the univariate case. Details are not presented here for the sake of brevity.

## Results

### Simulation overview

The accuracy of the new approximate confidence intervals isevaluatedfor a widerange of conditions. Appendix C: Simulation details contains more details of the simulations and examples. All simulations were conducted in SAS/IML (SAS 9.1, SAS Institute, 2003) using a version of LINMOD 3.4 modified to include the rank-adjusted Huynh-Feldt estimator and test. Predicted power values and approximate power confidence intervals were computed using a similarly modified version of POWERLIB 2.03. The modified versions of LINMOD and POWERLIB are available at *http://www.health-outcomes-policy.ufl.edu/muller/*.

### Simulation 1 with rank ***(X)=1*** (one-group repeated measures ANOVA)

The accuracy of the new approximate confidence intervals were evaluated for a completely within-subject design with *p*=9 repeated measures, *N*∈{10,20,40}, and *q*=rank(***X***)=1. Values for ***B***, contrast matrices ***C***, ***U***, and **Θ**_0_ were chosen to test a within-subject interaction for *α*=0.05. The model was chosen to ensure predicted power values for the Geisser-Greenhouse test of 0.20, 0.50, and 0.80, using the power approximation in Muller et al. [5]. Population covariance matrices were chosen to provide *∊*∈{0.282,0.505,0.720,1.00}. The sphericity values were selected to cover a range of eigenvalue patterns (i.e., patterns of the principal component variances) arising from the structure of **Σ**_∗_. For example, if *b*=3, then $\lambda ={\left[\begin{array}{lll} 1 & 0.12 & 0.12 \end{array}\right]}^{\prime }$ gives *∊*≈0.50. In turn, $\lambda ={\left[\begin{array}{lll} 1 & 0 & 0 \end{array}\right]}^{\prime }$gives *∊*=1/3≈0.33. Pseudo-random realizations of the error matrix, ***E***, were generated and tests were calculated. The observed mean power values for the four UNIREP tests were calculated and tabulated for 500,000 replications per condition.

For the conditions described above, additional pseudo-random realizations of the error matrix were generated using an estimating study with sample size, *N*_est_, of 10 and rank of ***X***, rank (***X***_est_), of 1 with 500,000 replications per condition for all four UNIREP tests. Corresponding estimated covariance matrices were calculated, as well as lower and upper bounds for power. Both one- and two-sided confidence intervals were evaluated with target coverages of 90% and 95%. The number of replications gave a standard error of estimated coverage probability less than or equal to 0.0003 for 1−*α*=0.95, and 0.0004 for 1−*α*=0.90, nearly guaranteeing 3 digits of accuracy. Only coverage of observed mean power values, and not predicted, was tabulated. The accuracy of the predicted power values, with respect to the observed, made it essentially redundant to consider both.

Only the worst case results for two-sided 95% confidence intervals are presented here. The worst cases occurred with the smallest sample size for the target study. Table 2 contains results for the Box conservative test with a target sample size of 10. For a wide range of sphericity values and target power values, the target 95% estimated coverage is consistently reached. The two cases in which the target coverage is not reached occur with large population sphericity and low power. Under these conditions, the Box conservative test would not be used in practice.

**Table 2:** **Target 95% CI (two-sided) estimated coverage *(×100) *** **of simulated population power for *N = 10***

**Test**	***∊***	**Power**	**Lower tail**	**Coverage**	**Upper tail**
	0.282	0.123	1.1	97.8	1.1
		0.535	1.9	97.0	1.1
		0.930	1.7	97.3	1.0
	0.505	0.054	0.1	97.3	2.6
		0.266	0.5	97.0	2.5
Box		0.690	1.1	97.0	1.9
	0.720	0.052	0.4	94.1	5.5
		0.227	0.6	96.8	2.6
		0.569	1.4	97.0	1.6
	1	0.023	0.6	85.1	14.3
		0.117	0.5	96.0	3.5
		0.350	0.8	97.8	1.4
	0.282	0.155	3.1	94.7	2.2
		0.585	2.6	95.6	1.8
		0.942	1.8	96.6	1.6
	0.505	0.162	5.4	87.7	6.9
		0.520	3.8	90.6	5.6
GG		0.870	2.6	92.4	5.0
	0.720	0.203	2.4	92.3	5.3
		0.539	2.6	94.1	3.3
		0.856	3.3	94.2	2.5
	1	0.161	0.7	95.6	3.7
		0.438	1.4	97.0	1.6
		0.751	2.7	96.2	1.1
	0.282	0.166	3.8	93.5	2.7
		0.602	2.8	95.2	2.0
		0.946	1.9	96.3	1.8
	0.505	0.210	8.2	82.9	8.9
		0.592	4.7	88.5	6.8
HF		0.902	2.9	90.9	6.2
	0.720	0.271	3.6	90.9	5.5
		0.631	3.4	93.3	3.3
		0.904	4.0	93.6	2.4
	1	0.224	0.8	96.7	2.5
		0.531	1.8	97.1	1.1
		0.821	3.2	95.9	0.9

Standard error of coverage probability ×100≈0.0003×100.

Table 2 also contains coverage results for the Geisser-Greenhouse and the rank-adjusted Huynh-Feldt tests. The target 95% estimated coverage is consistently reached for extreme sphericity values for both tests. For midrange sphericity values, the coverage fell below the target coverage from 0.8% to 7.3% for the Geisser-Greenhouse, and 1.4% to 12.1% for the rank-adjusted Huynh-Feldt. Coverage accuracy improved as the estimated power increased. In practice, lower power values are of little concern. For target power of 0.80 for the Geisser-Greenhouse test, the largest deviation from the target 95% estimated coverage was 2.6% for the Geisser-Greenhouse test and 4.1% for the rank-adjusted Huynh-Feldt test. Both occurred for the population sphericity value of 0.505.

Only a spherical case is appropriate to consider for the uncorrected test because otherwise the test will have inflated test size. Simulation results in Table 3 show that the approximation for the uncorrected test (with sphericity) always reached the target estimated coverage for the uncorrected test. The conservative bias could be eliminated by using optimal maximum likelihood estimates for the common variance and covariance (Morrison [21]), rather than the unstructured covariance estimate. Additional small changes are needed, associated with degrees of freedom, and corresponding to making all choices of *e*_1_ through *e*_5_ equal to 1.

**Table 3:** **Target 95% CI (Two-sided) estimated coverage *(×100) *** **of simulated population power for the uncorrected test *(∊ = 1.00)***

**N**	**Population power**	**Lower tail**	**Coverage**	**Upper tail**
10	0.238	0.5	97.5	2.0
	0.551	1.5	97.6	0.9
	0.835	3.2	96.1	0.7
20	0.215	0.8	97.3	1.9
	0.520	1.6	97.6	0.8
	0.814	3.1	96.2	0.7
40	0.207	0.9	97.2	1.9
	0.509	1.5	97.7	0.8
	0.806	3.0	96.3	0.7

Standard error of coverage probability ×100≈0.0003×100.

Although not presented here, in general, the accuracy of the coverage improved directly with increasing sample size, for all tests and conditions. The accuracy of the approximate confidence bounds for all four UNIREP tests also improved as the population sphericity increased.

### Simulation 2 with rank (***X***)***>1***

All of the simulations in the second example considered the condition of rank of ***X*** greater than 1. The cases used *p*=5 repeated measures, *N*∈{16,32,48}, and *q*=rank(***X***)∈{2,4,8,16}, corresponding to a three-, five-, nine-, and seventeen-group comparison, respectively. Appropriate fixed matrices of regression coefficients, ***B***, contrast matrices, ***C*** and ***U***, and **Θ**_0_ were chosen to test a within-subject interaction for a test size, *α*, of 0.05. The matrices were also chosen to ensure approximate target predicted power values for the rank-adjusted Huynh-Feldt test of 0.20, 0.50, and 0.80. Specific design matrices, ***X***, were defined. Population covariance matrices were chosen to provide specific population sphericity values, *∊*∈{0.282,0.505,0.720,1.00}. Observed mean power values were simulated and tabulated in a similar manner to that described in section ‘Simulation 1 with rank ***(X)=1*** (one-group repeated measures ANOVA)’.

Pseudo-random realizations of the error matrix were generated using an estimating study with sample size, *N*_est_, of 16 and rank of ***X***, rank (***X***_est_), of 4 with 500,000 replications per condition for all four UNIREP tests. Corresponding estimated covariance matrices were calculated, as well as lower and upper bounds for power using the methods presented in section ‘Approximate UNIREP power confidence intervals UNIREP power confidence intervals’. Approximate confidence interval coverage was defined as the proportion of the 500,000 simulated bound realizations that successfully covered the observed mean power values for each condition described above. Only coverage of observed mean power values, and not predicted, were tabulated. The accuracy of the predicted power values, with respect to the observed, made it essentially redundant to consider both. Both one- and two-sided confidence intervals were evaluated with target coverages of 90% and 95%.

In practical biomedical research, low power values are of little concern. Rarely will one have a power targeted below 0.70. Therefore, only the results for target power values of 0.80 will be presented and discussed. Power confidence interval coverage converged to the target coverage as sample size increased. Only the worst case results for two-sided 95% confidence intervals are presented here. The worst cases occurred with the smallest sample size for the target study, for a variety of population sphericity values and estimated population powers.

In Table 4, the observed mean population powers are presented for the four UNIREP tests for the population sphericity values and ranks of ***X*** considered for target rank-adjusted Huynh-Feldt power of 0.80 and sample size of 16 or 48. In general, as the population sphericity increased and rank of ***X*** increased, the observed mean power values for the Box conservative, the Geisser-Greenhouse, and the rank-adjusted Huynh-Feldt tests decreased. Only the Box conservative had severely biased power values as the population sphericity increased.

**Table 4:** **Simulated population power for target power *= 0.80, N = 16 * and rank (*X *) *= q***

**N**				**Simulated population power**				
		***∊=0.282***			***∊=0.505***			
	***q***	**Box**	**GG**	**HF**	**Box**	**GG**	**HF**	
16	2	0.779	0.811	0.817	0.561	0.778	0.809	
	4	0.763	0.797	0.805	0.510	0.762	0.802	
	8	0.753	0.787	0.799	0.455	0.736	0.796	
		***∊=0.720***			***∊=1.000***			
	***q***	**Box**	**GG**	**HF**	**Box**	**GG**	**HF**	**UN**
16	2	0.457	0.760	0.805	0.399	0.748	0.790	0.799
	4	0.355	0.740	0.801	0.255	0.724	0.787	0.801
	8	0.267	0.695	0.795	0.138	0.655	0.775	0.800
		***∊=0.282***			***∊=0.505***			
	***q***	Box	**GG**	**HF**	**Box**	**GG**	**HF**	
48	2	0.803	0.843	0.845	0.586	0.802	0.813	
	4	0.773	0.812	0.815	0.552	0.794	0.805	
	8	0.766	0.800	0.803	0.544	0.787	0.799	
	16	0.762	0.796	0.799	0.522	0.780	0.795	
		***∊=0.720***			***∊=1.000***			
	***q***	Box	**GG**	**HF**	**Box**	**GG**	**HF**	**UN**
48	2	0.500	0.792	0.806	0.455	0.785	0.797	0.800
	4	0.427	0.787	0.802	0.346	0.781	0.796	0.800
	8	0.402	0.781	0.799	0.280	0.778	0.797	0.801
	16	0.359	0.775	0.799	0.221	0.770	0.795	0.801

Standard error of estimated power ≈0.0006.

In Table 5, the proportion of simulations in which the estimated confidence interval successfully covered the observed mean population power values for each test is shown. The results are based on using an estimating study with sample size, *N*_est_, of 16 and rank of ***X***, rank (***X***_est_), of 4. In general, the approximate power confidence intervals nearly always reached the target 95% coverage for the Box conservative test. The coverage became more conservative as rank of ***X*** decreased. Similarly, the coverage became more conservative for the Geisser-Greenhouse and rank-adjusted Huynh-Feldt tests as rank of ***X*** decreased. The Geisser-Greenhouse and rank-adjusted Huynh-Feldt tests performed adequately in all cases except for the midrange population sphericity value, *∊*=0.505. The largest deviation from the target 95% estimated coverage was 13.6% and 16.0% for the Geisser-Greenhouse and rank-adjusted Huynh-Feldt tests, respectively, which occurred for *∊*=0.505 and rank of ***X*** equal to 8. The approximate power confidence intervals for the uncorrected test reached the target coverage value for every case considered in which the uncorrected test would be used.

**Table 5:** **Target 95% CI (two-sided) estimated coverage *(×100) *** **of simulated population power for target power *= 0.80, N = 16, *** **rank *(X) = q, *** **N**_**est**_ ***= 16, *** **and rank (*X***_**est**_**) *= 4***

	***∊* = 0.282**					*** ∊* = 0.505**		
***q***	**Box**	**GG**	**HF**		**Box**	**GG**	**HF**	
2	97.8	97.2	97.0		97.5	93.4	92.3	
4	93.7	92.0	91.6		95.6	86.8	85.0	
8	90.9	87.9	87.2		94.8	81.4	79.0	
	***∊=0.720***					*** ∊=1.000***		
	**Box**	**GG**	**HF**		**Box**	**GG**	**HF**	**UN**
2	97.6	95.4	94.9		97.4	95.3	95.5	95.8
4	97.5	93.6	92.9		97.6	96.8	97.0	97.4
8	96.9	90.6	89.8		97.0	96.1	96.9	97.4

Standard error of coverage probability × 100 ≈ 0.0003 × 100.

Although not presented here, in general, as sample size increased the conservative coverage values observed for the Box conservative and the uncorrected tests slowly converged to the target coverage value. This trend was observed for the conservative coverage values with the extreme population sphericity values for the Geisser-Greenhouse and the rank-adjusted Huynh-Feldt tests as well. The same is true of the liberal coverage values observed for the midrange population sphericity values for the Geisser-Greenhouse and the rank-adjusted Huynh-Feldt tests. Similar results were obtained for the target 90% two-sided confidence interval coverage as well as the 95% and 90% one-sided confidence intervals coverage.

The estimated coverages of these tabulated observed mean power values for each test were simulated for population sphericity values of 0.282, 0.505, 0.720, and 1.00. In Table 6, the worst case results from these simulations, which occurred for population sphericity 0.505, are presented. Approximate confidence intervals were simulated for 500,000 replications per condition (standard error of estimated coverage probability less than or equal to 0.0003 for 1−*α*=0.95, and 0.0004 for 1−*α*=0.90). The estimating studies use sample sizes, *N*_est_, of 16, 32, and 48, and ranks of ***X***_est_ of 2, 4, and 8.

**Table 6:** **Target 95% CI (two-sided) estimated coverage *(×100) * of simulated population power for *∊=0.505 *, target power *= 0.80, N= 48 * and rank *(X) = q***

		**Box coverage**			**GG coverage**			**HF coverage**		
		**rank *(X***_***est***_ ***)***			**rank *(X***_***est***_ ***)***			**rank *(X***_***est***_ ***)***		
***N***_***est***_	***q***	***2***	***4***	***8***	***2***	***4***	***8***	***2***	***4***	***8***
16	2	97.5	97.2	97.4	94.1	94.3	94.9	92.2	92.1	93.6
	4	94.8	94.8	95.3	87.3	87.5	88.9	85.9	86.2	87.4
	8	92.6	92.7	93.4	83.2	83.4	86.0	81.4	82.0	84.3
	16	92.0	92.3	93.7	82.3	82.5	85.9	80.5	80.7	83.2
32	2	97.3	97.3	97.3	93.3	93.3	93.4	92.4	92.5	92.6
	4	93.8	94.1	94.3	85.7	85.2	85.8	85.0	84.8	84.5
	8	91.6	91.8	91.4	81.3	81.5	82.4	79.5	80.0	80.6
	16	91.5	91.7	91.7	79.4	78.9	80.0	79.2	79.1	79.5
64	2	97.2	97.2	97.4	93.6	93.7	93.5	92.6	92.5	92.8
	4	94.4	94.6	94.8	84.5	85.0	84.7	84.4	85.0	85.2
	8	91.7	91.5	91.8	79.6	80.1	80.4	78.9	79.2	79.6
	16	90.9	90.9	91.0	78.5	78.4	78.7	78.9	78.4	78.7

Estimation Study: ***N***_***est***_***∈*** (16, 32, 48) and rank (***X***_***est***_***∈***) (2,4,8). Standard error of coverage probability × 100 ≈ 0.0003 × 100.

In general, for population sphericity values of 0.282 and 0.505, the approximate power confidence interval coverage for the Box conservative test converged to the target coverage value as rank of ***X***_est_ increased, and thus *ν*_est_ decreased. Coverage decreased as rank of ***X*** from the target study increased. For larger rank of ***X***, the approximate power confidence interval coverage fell short of the target coverage in several instances. No clear trend was apparent as *N*_est_ increased. The Box conservative test would not be used for larger population sphericity values. However, the realization that the target coverage was reached in nearly every case considered for the larger population sphericity values is worth mentioning.

The approximate power confidence interval coverages for both the Geisser-Greenhouse and rank-adjusted Huynh-Feldt tests seem to have converged to the target coverage value as rank of ***X***_est_ increased, and thus *ν*_est_ decreased, except in cases of sphericity. Such cases have little practical importance since exact results are available if sphericity is valid. Coverage decreased as rank of ***X*** from the target study increased. As observed in previous simulations, the approximate power confidence interval coverages for both the Geisser-Greenhouse and rank-adjusted Huynh-Feldt tests fell short of the target coverage to varying degrees in nearly every case considered for midrange population sphericity values. This outcome was also observed for larger rank of ***X*** from the target study for population sphericity of 0.282. The approximate power confidence interval coverage for the uncorrected tests reached the target coverage value in every case except for large *ν*_est_ and small rank of ***X*** from the target study. The approximate power confidence interval coverage increased as the ranks of ***X*** for both the target and estimating studies increased and as *N*_est_ decreased.

The slow convergence of the approximate power confidence interval coverage to the target coverage may be due, in part, to use of ${\tilde{∊}}_{n}$ and ${\tilde{∊}}_{r}$ in the approximate power confidence interval equation. These estimators of the sphericity parameter are ratios of unbiased estimators for the non-null and null cases, respectively. The variances of these estimators are much larger than the variances for ${\hat{∊}}_{n}$ and ${\hat{∊}}_{d}$. The larger variances may account for the slow convergence to the population power as the target and estimating study sample sizes and degrees of freedom increase. Further simulations may be needed to confirm this reasoning.

### Alternate approximations

In attempts to develop even better confidence bound estimates, additional approximations were developed and evaluated. One approach approximated the distribution of ${\tilde{\lambda }}_{*1}$ with an *F*. Using the methods presented in Kim et al. [22], the numerator of ${\tilde{\lambda }}_{*1}$ was approximated with a weighted noncentral chi-square, while the denominator was approximated with a weighted central chi-square. Two concerns arose. First, the denominator is not necessarily a central quadratic. The 2tr(***Δ***/*a*) component makes the denominator more of a shifted central quadratic. Second, the Kim et al. [22] result requires that the components of the numerator and denominator be mutually independent, which does not hold. Simulations demonstrated that the approximation was inaccurate in small samples.

Alternative approximations were developed and evaluated. The alternatives matched only the numerator to a weighted noncentral chi-square or to a weighted central chi-square with the denominator a constant equal to E$\left[\text{tr}({\hat{\Sigma }}_{*})+2\text{tr}(\Delta /a)\right]$. All were less accurate than the approximation presented here.

## Discussion

Even for small sample sizes, the proposed power confidence intervals attain very accurate coverage probabilities for the Box conservative test in all cases and for the uncorrected test with *∊*=1 (the only case for which it should be used). The result is also true for the extreme population sphericity values for the Geisser-Greenhouse and rank-adjusted Huynh-Feldt tests. For midrange population sphericity values, the coverage probabilities of the approximate power confidence intervals for the latter two tests often fell somewhat short of the various target coverage values considered. Coverage probabilities improve as sample size increases. Accuracy is better for higher target power values than for lower, which makes the results useful in practice. One-sided confidence intervals are recommended for lower bounds on power.

The techniques also allow plotting power confidence regions around an estimated power curve (Figure 1). The resulting plots have been well received by researchers.

## Conclusions

Good statistical practice requires associating a credible measure of uncertainty with any parameter estimate. We described and evaluated new methods to meet the need for UNIREP power estimates based on an estimated covariance with fixed means. Across a large range of conditions, the methods provide reasonably accurate coverage for all four UNIREP tests.

## Appendix

### Appendix A: Additional notation

The additional notation comes from Muller et al. [5], who showed that if $S_{t1}=\sum_{k=1}^{b}\lambda_{k}$, $S_{t2}=\sum_{k=1}^{b}\lambda_{k}^{2}$, $S_{t3}=\sum_{k=1}^{b}\lambda_{k}\omega_{k}$ and $S_{t4}=\sum_{k=1}^{b}\lambda_{k}^{2}\omega_{k}$, then

(A.1) $$\begin{array}{l} \lambda_{*1}=\frac{\left(aS_{t2}+2S_{t4}\right)}{\left(aS_{t1}+2S_{t3}\right)}\; \end{array}$$

(A.2) $$\begin{array}{l} \nu_{*1}=aS_{t1}/\lambda_{*1}\; \end{array}$$

(A.3) $$\begin{array}{l} \omega_{*}=\;S_{t3}/\lambda_{*1}\; \end{array}$$

(A.4) $$\begin{array}{l} \lambda_{*2}=\;S_{t2}/S_{t1}\; \end{array}$$

(A.5) $$\begin{array}{l} \nu_{*2}=\nu_{e}S_{t1}^{2}/S_{t2}=\nu_{e}\mathrm{b∊}\text{.}\; \end{array}$$

They used the parameters (assumed known) to approximate the UNIREP test statistic with a noncentral *F* distribution, as presented in equation 6.

## Appendix B: Supporting lemmas and proofs

### Lemma B.1

A.1-A.5 imply $\left\{S_{t1},S_{t2},S_{t3},S_{t4}\right\}=\;\left\{\text{tr}(\Sigma_{*}),\right)\left(\text{tr}(\Sigma_{*}^{2}),\text{tr}(\Delta ),\text{tr}(\Sigma_{*}\Delta )\right\}$.

### Proof of lemma B.1

$$\begin{array}{l} S_{t1}=\overset{b}{\underset{k=1}{\sum }}\lambda_{k}=\overset{b}{\underset{k=1}{\sum }}\text{tr}\;\left(\lambda_{k}\upsilon_{k}^{\prime }\upsilon_{k}\right)=\overset{b}{\underset{k=1}{\sum }}\text{tr}\;\left(\lambda_{k}\upsilon_{k}\upsilon_{k}^{\prime }\right)\\ \;=\text{tr}\left[\overset{b}{\underset{k=1}{\sum }}\left(\lambda_{k}\upsilon_{k}\upsilon_{k}^{\prime }\right)\right]=\text{tr}\;\left(\Sigma_{*}\right)\\ S_{t2}=\overset{b}{\underset{k=1}{\sum }}\lambda_{k}^{2}=\overset{b}{\underset{k=1}{\sum }}\text{tr}\;\left(\lambda_{k}^{2}\upsilon_{k}^{\prime }\upsilon_{k}\right)=\overset{b}{\underset{k=1}{\sum }}\text{tr}\;\left(\lambda_{k}^{2}\upsilon_{k}\upsilon_{k}^{\prime }\right)\\ \;=\text{tr}\;\left[\overset{b}{\underset{k=1}{\sum }}\left(\lambda_{k}^{2}\upsilon_{k}\upsilon_{k}^{\prime }\right)\right]=\text{tr}\;\left(\Sigma_{*}^{2}\right)\\ S_{t3}=\overset{b}{\underset{k=1}{\sum }}\text{tr}\;\left(\lambda_{k}\frac{v_{k}^{\prime }{\Delta \upsilon }_{k}}{\lambda_{k}}\right)=\overset{b}{\underset{k=1}{\sum }}\text{tr}\;\left({\Delta \upsilon }_{k}\upsilon_{k}^{\prime }\right)\\ \;=\text{tr}\;\left[\Delta \overset{b}{\underset{k=1}{\sum }}\left(\upsilon_{k}\upsilon_{k}^{\prime }\right)\right]=\text{tr}\;\left({\Delta \Upsilon \Upsilon }^{\prime }\right)=\text{tr}\left(\Delta \right)\\ S_{t4}=\overset{b}{\underset{k=1}{\sum }}\text{tr}\;\left(\lambda_{k}^{2}\frac{v_{k}^{\prime }{\mathrm{\Delta v}}_{k}}{\lambda_{k}}\right)=\overset{b}{\underset{k=1}{\sum }}\text{tr}\;\left(\Delta \lambda_{k}v_{k}v_{k}^{\prime }\right)\\ \;=\text{tr}\;\left[\Delta \overset{b}{\underset{k=1}{\sum }}\left(\lambda_{k}v_{k}v_{k}^{\prime }\right)\right]=\text{tr}\;\left({\Delta \Sigma }_{*}\right)\text{.} \end{array}$$

### Lemma B.2

The first moments of $\text{tr}({\hat{\Sigma }}_{*})$, $\text{tr}({\hat{\Sigma }}_{*}^{2})$, ${\text{tr}}^{2}({\hat{\Sigma }}_{*})$, and $\text{tr}(\Delta {\hat{\Sigma }}_{*})$ are known.

### Proof of lemma B.2

Following Wishart [23], $S=\nu_{e}{\hat{\Sigma }}_{*}∼W_{b}\left(\nu_{e},\Sigma_{*}\right)$, such that *ν*_*e*_=*N*−rank(***X***). For Wishart ${\langle \Sigma \rangle }_{\text{jj}}=\sigma_{j}^{2}$ while here 〈**Σ**〉_*jj*_=*σ*_*jj*_ and *ρ*_*jk*_=*σ*_*jk*_/(*σ*_*jj*_*σ*_*kk*_)^1/2^. Wishart [23] said, with emphasis not in the original, “ …moment coefficients are in all cases *except the first* calculated about the mean of the sample …”. Here *μ*(*n*) indicates the expression in equation *n* at the end of Wishart [23], $\text{E}\left[\text{tr}\left(S\right)\right]=\text{E}(\sum_{{j=1}^{b}}s_{\text{jj}})=\sum_{j=1}^{b}\text{E}\left(s_{\text{jj}}\right)=\sum_{j=1}^{b}\mu {\left(1\right)}_{j}=\sum_{j=1}^{b}\nu_{e}\sigma_{\text{jj}}$. Thus

(B.1) $$\begin{array}{ll} \begin{array}{l} \text{E}[\text{tr}({\hat{\Sigma }}_{*})]=\left(1/\nu_{e}\right)\text{E}\left[\text{tr}\left(S\right)\right]=\left(1/\nu_{e}\right)\overset{b}{\underset{j=1}{\sum }}\nu_{e}\sigma_{\text{jj}}=\text{tr}\left(\Sigma_{*}\right). \end{array} & \; \end{array}$$

With $S^{2}={\left(\nu_{e}{\hat{\Sigma }}_{*}\right)}^{2}$, $\text{E}\left[\text{tr}(S^{2})\right]=\text{E}\left[\left(\sum_{j=1}^{b}\sum_{k=1}^{b}s_{\text{jk}}^{2}\right)\right]=\sum_{j=1}^{b}\sum_{k=1}^{b}\text{E}\left(s_{\text{jk}}^{2}\right)$. In turn $\text{E}\left(s_{\text{jk}}^{2}\right)=\text{E}\left\{{\left[\left[s_{\text{jk}}-\text{E}\left(s_{\text{jk}}\right)\right]+\text{E}\left(s_{\text{jk}}\right)\right]}^{2}\right\}=\text{E}\left\{{\left[s_{\text{jk}}-E\left(s_{\text{jk}}\right)\right]}^{2}+2\text{E}\left(s_{\text{jk}}\right)\left[s_{\text{jk}}-E\left(s_{\text{jk}}\right)\right]+{\left[\text{E}\left(s_{\text{jk}}\right)\right]}^{2}\right\}=\text{E}\left\{{\left[s_{\text{jk}}-E\left(s_{\text{jk}}\right)\right]}^{2}\right\}+2\text{E}\left(s_{\text{jk}}\right)\text{E}\left[s_{\text{jk}}-E\left(s_{\text{jk}}\right)\right]+{\left[\text{E}\left(s_{\text{jk}}\right)\right]}^{2}=\text{E}\left\{{\left[s_{\text{jk}}-E\left(s_{\text{jk}}\right)\right]}^{2}\right\}+{\left[\text{E}\left(s_{\text{jk}}\right)\right]}^{2}$. Also:

$$\text{E}\left(s_{\text{jk}}^{2}\right)=\left\{\begin{array}{ll} \left[s_{\text{jj}}^{2}-{\left[\text{E}\left(s_{\text{jj}}\right)\right]}^{2}\right]+{\left[\text{E}\left(s_{\text{jj}}\right)\right]}^{2}=\mu {\left(3\right)}_{j}+{\left[\mu {\left(1\right)}_{j}\right]}^{2}=\nu_{e}\sigma_{\text{jj}}^{2}\left(2+\nu_{e}\right) & \;\text{if}j=k\\ \left[s_{\text{jk}}^{2}-{\left[\text{E}\left(s_{\text{jk}}\right)\right]}^{2}\right]+{\left[\text{E}\left(s_{\text{jk}}\right)\right]}^{2}=\mu {\left(5\right)}_{\text{jk}}+{\left[\mu {\left(2\right)}_{\text{jk}}\right]}^{2}=\nu_{e}\left(\sigma_{\text{jj}}\sigma_{\text{kk}}+\sigma_{\text{jk}}^{2}\right) & \;\text{if}j\neq k\text{.} \end{array}\right)$$

Hence

(B.2) $$\text{E}[\text{tr}({\hat{\Sigma }}_{*}^{2})]=\left(1/\nu_{e}^{2}\right)\left[\nu_{e}\left(\nu_{e}+1\right)\text{tr}(\Sigma_{*}^{2})+\nu_{e}{\text{tr}}^{2}\left(\Sigma_{*}\right)\right]\text{.}$$

Here $\text{E}\left[{\text{tr}}^{2}\left(S\right)\right]=\text{E}\left[\text{tr}\left(S\right)\text{tr}\left(S\right)\right]=\text{E}\left[\left(\overset{b}{\underset{j=1}{\sum }}s_{\text{jj}}\right)\left(\overset{b}{\underset{k=1}{\sum }}s_{\text{kk}}\right)\right]=\text{E}\left(\sum_{j=1}^{b}\sum_{k=1}^{b}s_{\text{jj}}s_{\text{kk}}\right)=\sum_{j=1}^{b}\sum_{k=1}^{b}\text{E}\left(s_{\text{jj}}s_{\text{kk}}\right)$, with $\text{E}\left[s_{\text{jj}}s_{\text{kk}}\right]=\mu {\left(4\right)}_{\text{jk}}=2\nu_{e}\sigma_{\text{jk}}^{2}+\nu_{e}^{2}\sigma_{\text{jj}}\sigma_{\text{kk}}$. Thus,

(B.3) $$\begin{array}{lll} \text{E}\left[{\text{tr}}^{2}({\hat{\Sigma }}_{*})\right] & =\left(1/\nu_{e}^{2}\right)\text{E}\left[{\text{tr}}^{2}\left(S\right)\right]\; & \;\\ & =\left(1/\nu_{e}^{2}\right)\sum_{j=1}^{b}\sum_{k=1}^{b}\left(2\nu_{e}\sigma_{\text{jk}}^{2}+\nu_{e}^{2}\sigma_{\text{jj}}\sigma_{\text{kk}}\right)\; & \;\\ & =\left(1/\nu_{e}^{2}\right)[2\nu_{e}\text{tr}({\hat{\Sigma }}_{*}^{2})+\nu_{e}^{2}{\text{tr}}^{2}\left(\Sigma_{*}\right)]\text{.}\; \end{array}$$

Finally, $S=\nu_{e}{\hat{\Sigma }}_{*}∼W_{b}\left(\nu_{e},\Sigma_{*}\right)$ has E (***S***)=*ν*_*e*_**Σ**_∗_ (Muller and Stewart [6], Theorem 10.10). Hence E [tr(***ΔS***)]=tr[E(***ΔS***)]=tr[***Δ***E(***S***)]=tr[***Δ***(*ν*_*e*_**Σ**_∗_)]=*ν*_*e*_tr(***ΔΣ***_∗_) and

(B.4) $$\text{E}\left[\text{tr}(\Delta {\hat{\Sigma }}_{*})\right]=\text{tr}({\Delta \Sigma }_{*}).$$

### Proof of lemma 1

Substituting equivalent terms from equations A.1-A.5 into (*λ*_∗2_*abν*_∗2_)/(*λ*_∗1_*ν*_∗1_*bν*_*e*_) allows combining like terms and simplifying to give

$$\frac{\lambda_{*2}}{\lambda_{*1}}\frac{\text{ab}}{\nu_{*1}}\frac{\nu_{*2}}{b\nu_{e}}=\frac{S_{t2}/S_{t1}}{\lambda_{*1}}\frac{\text{ab}}{aS_{t1}/\lambda_{*1}}\frac{\nu_{e}S_{t1}^{2}/S_{t2}}{b\nu_{e}}=1\text{.}$$

If $T_{u}=[\text{tr}(\hat{\Delta })/)a]/[\text{tr}({\hat{\Sigma }}_{*})]$, then $\text{tr}(\hat{\Delta })\approx \lambda_{*1}y_{*1}$ and $\nu_{e}\text{tr}({\hat{\Sigma }}_{*})\approx \lambda_{*2}y_{*2}$ with *y*_∗1_∼*χ*^2^(*ν*_∗1_,*ω*_∗_) and *y*_∗2_∼*χ*^2^(*ν*_∗2_) as described in Muller et al. [5]. In turn,

$$\mathrm{Pr}\left\{T_{u}\leq t\right\}=\mathrm{Pr}\left\{[\text{tr}(\hat{\Delta })/)a]/\text{tr}({\hat{\Sigma }}_{*})\leq t\right\}\approx \mathrm{Pr}\left\{\left[\lambda_{*1}y_{*1}/(\text{ab})\right]/\left[\lambda_{*2}y_{*2}/\left(b\nu_{e}\right)\right]\leq t\right\}=$$

Pr{(*y*_∗1_/*ν*_∗1_)/(*y*_∗2_/*ν*_∗2_)≤*tλ*_∗2_*abν*_∗2_/(*λ*_∗1_*ν*_∗1_*bν*_*e*_)}=*F*_*F*_(*t*;*ν*_∗1_,*ν*_∗2_,*ω*_∗_).

### Proof of lemma 2

Using Lemma B.2, unbiased estimators for tr^2^(**Σ**_∗_) and $\text{tr}(\Sigma_{*}^{2})$ are ${\hat{\tau }}_{1}=\;[{\text{tr}}^{2}({\hat{\Sigma }}_{*})-2{\left(\nu_{e}+1\right)}^{-1}\text{tr}({\hat{\Sigma }}_{*}^{2})]{\left\{1-2{\left[\nu_{e}\left(\nu_{e}+1\right)\right]}^{-1}\right\}}^{-1}$ and ${\hat{\tau }}_{2}=[\nu_{e}^{2}\text{tr}({\hat{\Sigma }}_{*}^{2})-\nu_{e}{\text{tr}}^{2}({\hat{\Sigma }}_{*})]{\left[\nu_{e}\left(\nu_{e}+1\right)-2\right]}^{-1}$, as introduced in Gribbin [18] and Chi et al. [19]. As shown in Lemma B.2, $\text{tr}({\hat{\Sigma }}_{*})$ and $\text{tr}({\hat{\Sigma }}_{*}\Delta )$ are unbiased estimators for $\text{tr}({\hat{\Sigma }}_{*})$ and $\text{tr}({\hat{\Sigma }}_{*}\Delta )$, respectively. Thus,

$$\;\;\;{\tilde{∊}}_{n}=\frac{{\hat{\tau }}_{1}+2\text{tr}({\hat{\Sigma }}_{*})\text{tr}\left(\Delta /a\right)}{b\left[{\hat{\tau }}_{2}+2\text{tr}({\hat{\Sigma }}_{*}\Delta /a)\right]}=\frac{\nu_{e}\left(\nu_{e}+1\right){\text{tr}}^{2}({\hat{\Sigma }}_{*})-2\nu_{e}\text{tr}({\hat{\Sigma }}_{*}^{2})+2\left[\nu_{e}\left(\nu_{e}+1\right)-2\right]\text{tr}({\hat{\Sigma }}_{*})\text{tr}\left(\Delta /a\right)}{b\left\{\nu_{e}^{2}\text{tr}({\hat{\Sigma }}_{*}^{2})-\nu_{e}{\text{tr}}^{2}({\hat{\Sigma }}_{*})+2\left[\nu_{e}\left(\nu_{e}+1\right)-2\right]\text{tr}({\hat{\Sigma }}_{*}\Delta /a)\right\}}\text{.}$$

In the null case ***Δ***=***0*** and ${\tilde{∊}}_{n}$ reduces to the rank-adjusted sphericity estimator, ${\tilde{∊}}_{r}=\left[\left(\nu_{e}\;+\;1\right)\right)\left(\;b\hat{∊}-2\right]/$$\left[b\left(\nu_{e}-b\hat{∊}\right)\right]={\tilde{∊}}_{n}|\Delta =0$.

## Appendix C: Simulation details

### Covariance conditions

Covariance conditions 5-8 from Table III of Coffey and Muller [24] were used for each example described below: **Σ**_∗_=Dg(***λ***_*j*_) for *j*∈{1,2,3,4}, with

$$\begin{array}{ll} \lambda_{1}^{\prime }=[0.47960\;0.01000\;0.01000\;0.01000]\;, & \;\\ \lambda_{2}^{\prime }=[0.34555\;0.06123\;0.05561\;0.04721]\;, & \;\\ \lambda_{3}^{\prime }=[0.23555\;0.17123\;0.05561\;0.04721]\;, & \;\\ \lambda_{4}^{\prime }=[0.12740\;0.12740\;0.12740\;0.12740]\;. & \; \end{array}$$

Thus, *∊*∈{0.28,0.51,0.72,1.00}. Given **Σ**_∗_=Dg(***λ***_*j*_), it follows that **Σ**=***UΣ***_∗_***U***^′^.

### CLAHE mammography example

Computer scientists developed the Contrast Limited Adaptive Histogram Equalization (CLAHE) algorithm to improve contrast in medical images. Independent observers considered 3×3=9 Clip ×Region combinations. Region denotes the size of the image (pixels^2^) at which contrasts are controlled and Clip level limits the maximum contrast adjustment. In the multivariate model ***X***=***1***_*N*_, while within-person factors Clip and Region gave ***Y***, (*N*×9). Also ***B***, (1×9), contained mean log10(contrast) for the unprocessed condition minus the mean for each of the nine combinations of Clip and Region (*β*_cr_=*μ*_unprocessed_−*μ*_cr_). If ***T***_c_ contains orthonormal linear and quadratic trends for log2(Clip) ∈ {1,2,4}, and ***T***_r_ does the same for log2(Region) ∈ {1,3,5}, then the 9 × 4 within-persons contrast matrix, ***U***_cr_ is

$$U_{\text{cr}}=T_{c}⊗T_{r}=\left[\begin{array}{ll} -4/\sqrt{42} & 2/\sqrt{14}\\ -1/\sqrt{42} & -3/\sqrt{14}\\ 5/\sqrt{42} & 1/\sqrt{14} \end{array}\right]⊗\left[\begin{array}{ll} -1/\sqrt{2} & 1/\sqrt{6}\\ 0\;\;\;\;\;\;\;\;\;\;\;\; & -2/\sqrt{6}\\ 1/\sqrt{2} & 1/\sqrt{6} \end{array}\right]\text{.}$$

With L the linear and Q the quadratic trends for interaction components being tested, $U_{\text{cr}}=\left[\begin{array}{llll} u_{\text{LL}} & u_{\text{LQ}} & u_{\text{QL}} & u_{\text{QQ}} \end{array}\right]$.

All four covariance patterns were factorially combined with *N*∈{10,20,40}. The multivariate test considered $\Theta_{\text{cr}}=\beta_{P}\cdot \left[\begin{array}{llll} 0.5 & 1.0 & -1.0 & 0.5 \end{array}\right]$ with *α*=0.05, and *β*_*P*_ the scaling factor for ***B*** corresponding to approximate target power *P*∈{0.20,0.50,0.80} for the Geisser-Greenhouse approximation using methods in Muller et al. [5]. The conditions in the example were used in section ‘Simulation 1 with rank ***(X)=1*** (one-group repeated measures ANOVA)’. More details of the example are in Muller et al. [5].

### Test of interaction with rank (***X***)***>1*** Example

All cases used 5 repeated measures, *N*∈{16,32,48}, and rank (***X***)∈{2,4,8,16}. For obvious reasons, a rank of ***X*** equal to 16 was not considered for the smallest sample size. All four covariance patterns were factorially combined with the sample sizes and ranks ***X***. In the multivariate model, ***X***=***I***_*q*_⊗***1***_*repn*_, such that *repn* = *N*/*q*, and ⊗ is a Kronecker product. If

$$D_{16}=\left[\begin{array}{cc} \underset{q\times 5}{D_{a}} & \underset{q\times 11}{D_{b}}\\ \underset{\left(16-q\right)\times 5}{D_{c}} & \underset{\left(16-q\right)\times 11}{D_{d}} \end{array}\right]\text{,}$$

then ***B***=*β*_*P*_·***D***_*a*_, such that *β*_*P*_ was the scaling factor giving approximate target power *P*∈{0.20,0.50,0.80}, for the rank-adjusted Huynh-Feldt power approximation. The within-subject contrast, ***U***, (5×4), contained orthonormal linear, quadratic, cubic and quartic trends:

$$U=\left[\begin{array}{llll} -2/\sqrt{10} & 2/\sqrt{14} & -1/\sqrt{10} & 1/\sqrt{70}\\ -1/\sqrt{10} & -1/\sqrt{14} & 2/\sqrt{10} & -4/\sqrt{70}\\ 0/\sqrt{10} & -2/\sqrt{14} & 0/\sqrt{10} & 6/\sqrt{70}\\ 1/\sqrt{10} & -1/\sqrt{14} & -2/\sqrt{10} & -4/\sqrt{70}\\ 2/\sqrt{10} & 2/\sqrt{14} & 1/\sqrt{10} & 1/\sqrt{70} \end{array}\right]\text{.}$$

Each row of the between-subject contrast, ***C***, a (*q*−1×*q*) orthonormal matrix, contained one of the (*q*−1) trends. The contrasts define a test of interaction of between- and within-subject trends. Without loss of generality, we assumed **Θ**_0_=***0***. A test size, *α*, of 0.05 was used.

### Computational methods

All power computations were conducted in SAS/IML (SAS 9.1, SAS Institute, 2003). Free software LINMOD 3.4 was used for all data analysis and includes new methods. Free software POWERLIB 2.1 in Johnson et al. [25] was used for all power analysis and includes the new methods. Both are available at *http://health-outcomes-policy.ufl.edu/faculty-directory/-muller-keith/list-of-software/*. UNIREP power is also available in GLIMMPSE, a free web-browser based program with a graphical user interface aimed at health scientists (*http://www.SampleSizeShop.org*). The next version of GLIMMPSE is expected to implement the confidence interval methods.

## Competing interests

The authors declare that they have no competing interests.

## Authors’ contributions

The majority of the work comes directly from the dissertation of MJG at UNC Chapel Hill, in partial fulfillment of the requirements of the DrPH, under the direction of KEM. Dr. KEM and Dr. Y-YC assisted in revising and compressing the notation, text and tables. Dr. PWS was a member of the dissertation committee and assisted in formulating the proof of simultaneous coverage of the confidence regions and in final editing. All authors read and approved the final manuscript.

## Pre-publication history

The pre-publication history for this paper can be accessed here:

http://www.biomedcentral.com/1471-2288/13/57/prepub
